# Nutri-Score: Awareness, Perception and Self-Reported Impact on Food Choices among French Adolescents

**DOI:** 10.3390/nu14153119

**Published:** 2022-07-29

**Authors:** Pauline Ducrot, Chantal Julia, Anne-Juliette Serry

**Affiliations:** 1 Santé Publique France, French National Public Health Agency, 94415 Saint-Maurice, France; anne-juliette.serry@santepubliquefrance.fr; 2 Sorbonne Paris Nord University, Inserm U1153, Inrae U1125, Cnam, Nutritional Epidemiology Research Team (EREN), Epidemiology and Statistics Research Center—University of Paris (CRESS), 93000 Bobigny, France; julia@eren.smbh.univ-paris13.fr; 3 Department of Public Health, Hôpitaux Universitaires Paris Seine-Saint-Denis (AP-HP), 93000 Bobigny, France

**Keywords:** front-of-pack labeling, Nutri-Score, awareness, food choices, adolescents

## Abstract

To date, no studies have evaluated the appropriation of the front-of-pack Nutri-Score labeling among adolescents, although they are both consumers and buyers of food products. Therefore, the objectives of the present study were (1) to assess Nutri-Score awareness, perception and self-reported impact on food choices in French adolescents and (2) to identify the determinants associated with higher Nutri-Score awareness and self-reported impact on food choices. A web-based survey was conducted in November 2021 among 1201 adolescents. Multivariate logistic models were used to evaluate the relationships between individual factors and Nutri-Score awareness and self-reported impact on food choices. Almost all the adolescents reported to know the Nutri-Score (97.0%) and more than 9 out of 10 considered this logo easy to understand and easy to identify on food packages. Finally, 54% self-reported that the label had already impacted their food choices. Girls (2.28 (1.09–4.77), *p* = 0.028) and the 15–17-year-olds (3.12 (1.32–7.35), *p* = 0.0094) were more likely to be aware of the label compared with their respective counterparts (i.e., boys and the 11–14-year-olds). Regarding the impact of food choices, the use of the Nutri-Score by the parents was the most determinant criterion (7.74 (5.74–10.42), *p* < 0.0001). Thus, promotion campaigns should target both adolescents and parents.

## 1. Introduction

In order to tackle the burden of non-communicable chronic diseases related to unhealthy diets, the World Health Organization recommends, among other measures, the implementation of front-of-pack nutrition labeling (FoPL). First, such labels have the potential to help consumers make healthier food choices, by making the mandatory nutritional declaration accessible at the time of purchase, i.e., visible (at the front of pack) and understandable (with simplified and/or color-coded information). Furthermore, nutrition labels incentivize reformulations from food manufacturers, thereby improving the food offer [1,2].

Different types of labels are used worldwide; they can be divided into nutrient specific and summary formats. The latter gives an overall appraisal of the nutritional quality of the product and has therefore been demonstrated to be easier to interpret and to use by consumers [3,4]. The Nutri-Score belongs to this category: it rates the nutritional quality of food products on a five-color coded graded scale associated with letters to improve readability (ranging from A “dark green” for “higher nutritional quality”, to E “dark orange” for “lower nutritional quality”). The classification is based on a modified Food Standard Agency’s/OfCom nutrient profiling system, adapted to the French context [5].

After its first implementation in France in 2017, a number of other European countries have also adopted the Nutri-Score: Belgium, Switzerland, Luxembourg, Germany, Spain and the Netherlands.

The logo and its underlying algorithm have been the subject of numerous studies at the French and European levels. Its effectiveness in guiding consumers towards products of better nutritional quality has been widely demonstrated [6,7,8,9,10,11,12,13], as well as the algorithm’s prospective associations with different health indicators [14,15,16,17,18,19,20].

Nevertheless, to our knowledge, the studies conducted so far have involved adults and none have evaluated the potential effectiveness of Nutri-Score on adolescents. However, the latter group represents a target of interest insofar as they are both consumers and buyers of food products and they are likely to strongly influence the purchasing decisions of their parents [21]. In addition, young people are often the target of marketing for products of which consumption should be limited [22], while they do not always have sufficiently developed critical thinking capacities (insufficient literacy in advertising), thus making them more vulnerable to these techniques [23]. Finally, given the growing risk of obesity and diseases associated with nutritional factors, children and adolescents are considered a priority target for nutritional prevention actions. Indeed, in addition to being a risk factor by itself for overweight or obesity in adults [24], childhood obesity is associated with an increased risk of comorbidities such as diabetes, cancer, cardiovascular disease and premature mortality [25,26,27].

For these reasons, it is of importance to evaluate the appropriation of FOP nutrition labels by adolescents. The objectives of the present study were to evaluate the Nutri-Score awareness, perception and self-reported impact on food choices in French adolescents. In addition, this study aims at identifying the individual factors associated with higher Nutri-Score awareness and self-reported impact on food choices.

## 2. Materials and Methods

### 2.1. Population Study

A national and representative sample of 1201 French adolescents (11–17 years old) was recruited through an access panel on the internet in November 2021. An e-mail was sent to a panel (*N* = 32,715) of parents of adolescents or adolescents directly (if aged ≥16) to invite them to participate in a study regarding food behaviors, with no mention of FoPL. 

The questionnaire included questions on individual characteristics of adolescents (gender, age, size of urban area, region) and occupational category of the reference person in the household (categorized as follows: upper category: head of businesses, managerial staff, intermediate profession; lower category/inactive: employees, laborers, students, retired, unemployed). In addition, data regarding the perception of their weight status (too skinny, normal weight, overweight) were also collected. The questionnaire used in the study (translated in English) is available as Supplementary material. The total completion time was about 6 min (including the parts described in the following sections).

### 2.2. General Purchasing Behavior

The questionnaire also included questions to assess how often adolescents ask their parents to buy specific food products, go food purchasing with their parents and buy foods alone. Three modalities of answer were proposed: often, sometimes, never. 

### 2.3. Awareness, Perception and Purchasing Behavior Regarding Nutri-Score

The awareness of the logo was assessed through two questions: “have you ever heard of the Nutri-Score logo?” (Yes/No) and the recognition of the logo after presenting the visual to the participant. Adolescents were considered to be aware of the Nutri-Score if they answered “yes” to at least one of the two questions.

The perception of the logo was assessed through two statements: “this logo is easy to understand” and “this logo is easy to identify on food packages” (the latter was assessed among participants who reported having already heard of or seen the Nutri-Score). Answers were collected using a 4-point Likert scale (from fully agree to fully not agree) and recoded as “yes”/"no”. 

Then, the self-reported impact of the logo on purchasing behaviors was evaluated among participants who reported knowing the Nutri-Score by asking them how often they purchase food products with the Nutri-Score (often, sometimes, never, I did not see the logo during my food purchase, I do not know). Participants who had already bought a product with a Nutri-Score logo on it were also asked whether it had been an incentive to buy the product (yes/no). 

In addition, participants who declared having already heard of or seen the Nutri-Score were asked if they changed their purchasing behavior according to the logo (often, sometimes, never):

- Choice of a product with the logo, instead of another product without the logo;
- Choice of a product or a brand with a better classification (e.g., choosing a product with a Nutri-Score D instead of E);
- Asking their parents to buy a specific product on which the Nutri-Score was affixed.

Finally, questions were asked regarding the parents’ purchasing behaviors. Adolescents were asked whether their parents take into account the Nutri-Score during their food purchase (often, sometimes or never) and whether they have ever accepted to buy them a specific product because its Nutri-Score was A or B or on the contrary refused to buy a product because the Nutri-Score was D or E (yes/no).

### 2.4. Ethical Considerations

The access panel was implemented by a marketing survey firm specialized in opinion polls: the BVA Group. The questionnaire was administered to eligible respondents according to BVA Group’s ethical procedures. Participation in this study was on a voluntary basis. An electronic informed consent was obtained from each participant before starting the questionnaire. Respondents had the possibility to interrupt their participation at any time. They were given small incentives for participating and were compensated in the form of points, which they can accumulate over time and convert into gifts of different types.

The personal data treatment of participants was carried out in accordance with the French law n°78–17 of the 6 January 1978 and the European regulation n°2016/679, known as the General Data Protection Regulation. 

### 2.5. Statistical Analyses

The representative nature of the sample was ensured by using the quota method based on the following variables: gender, age, occupational category of the participant and the reference person in the household, region and agglomeration size. The structure for adjustment came from data from the 2017 national census of the national French Statistics Institute (Insee), which was also used for weighting the data. 

Analyses were stratified by age (11–14/15–17). Chi-square tests were used to compare individual characteristics, general purchasing practices and awareness, perception and purchasing practices related to the Nutri-Score label across age categories. Logistic regression models were used to assess the relationship between awareness of the Nutri-Score and individual characteristics. A similar model was used for the self-reported impact on purchasing behavior. For the latter, the outcome was having changed at least one purchasing behavior thanks to the Nutri-Score among the three behaviors evaluated. The common individual characteristics used in the models were gender, age, occupational category of the reference person in the household, perceived weight status and buying food alone or not. The model on behaviors was also adjusted on the use of the Nutri-Score by the parents (data access only in adolescents who reported knowing the Nutri-Score). 

The statistical significance level was set at 5%. Analyses were carried out with the S.A.S software (Version 7.11; SAS Institute, Inc., Cary, NC, U.S.). 

## 3. Results

### 3.1. Population Study

The individual characteristics of the sample are presented in Table 1. The total sample comprised a similar proportion of boys and girls (51% and 49%, respectively). About 57% were 11–14 years old (vs. 43% for the 15–17-year-olds). Among the younger, the proportion of boys was higher (*p* < 0.005). The occupational category of the reference person in the household was well balanced, with 49% in the upper category and 51% in the lower category or inactive. Finally, 83% of the sample reported to consider having a normal weight.

### 3.2. Purchasing Practices of Adolescents on Foods and Beverages

The purchasing practices of adolescents on foods and beverages are presented in Table 2.

Almost all the adolescents (94.4%) reported asking their parents to buy specific foods or beverages products sometimes or often. They were also largely involved in food purchases, with 8 adolescents out of 10 going for food purchases with their parents or another person (sometimes or often) and more than 1 out of 2 buying food or beverages on their own. The older participants (15–17) were more likely to do so (*p* < 0.0001), with 72.8% buying food alone, whereas among the younger (11–14) only 42.6% reported to do so.

### 3.3. Awareness, Perception and Purchasing Practices Related to the Nutri-Score

The awareness, perception and purchasing practices related to the Nutri-Score label are presented in Table 3. Almost all the adolescents reported knowing the Nutri-Score, with 97.0% of them having already heard or seen the logo and even more among the 15–17-year-olds (98.6% vs. 95.8%, *p* = 0.004).

The perception of the logo is also very favorable, with more than 9 adolescents out of 10 considering the Nutri-Score easy to understand and easy to identify.

As regards food purchase, 7 out of 10 declared they had already bought a product with the Nutri-Score, in particular the 15–17-year-olds (77.9% vs. 67.0%, *p* < 0.0001). The logo was an incentive for buying the product for about 54% of these adolescents and again the 15–17-year-olds were more likely to be influenced, with 58.6% who reported they have bought a product due to the Nutri-Score vs. 49.4% of the 11–14-year-olds (*p* = 0.008).

More specifically, the older participants were again more likely to report a change in purchasing behavior, except for asking their parents to buy a specific product because the Nutri-Score is affixed on it. Indeed, only 15% of the participants reported they have ever asked for a product from their parents for this reason, but without significant difference between age group. However, this behavior was relatively rare, with only 15% of the adolescents reporting a change on the latter.

Finally, regarding parents’ behavior, participants reported that a majority of their parents take into account the Nutri-Score during food purchases (7 out of 10) and that the label can influence their responses to the adolescents’ demand for some food products. However, according to the adolescents, parents were more likely to accept to buy a product because its Nutri-Score is A or B than to refuse because its Nutri-Score is D or E.

### 3.4. Nutri-Score Awareness and Change in Purchasing Behavior: Associations with Individual Characteristics

In Table 4 are presented the results of the multivariate regression logistic models showing the relationship between awareness and individual characteristics on one hand and the change in purchasing behavior and individual characteristics on the other hand.

The Nutri-Score awareness was significantly higher in girls (2.28 (1.09–4.77), *p* = 0.028) and in the 15–17-year-olds (3.12 (1.32–7.35), *p* = 0.0094).

As regards change in purchasing behavior, the Nutri-Score was more likely to influence adolescents whose parents also take into account the logo during their grocery shopping (7.74 (5.74–10.42), *p* < 0.0001), while the adolescents who perceived themselves as too skinny were less likely to be affected (0.56 (0.35–0.89), *p* = 0.030).

## 4. Discussion

This study provides new data on the awareness, perception and self-reported use of the Nutri-Score in France by adolescents and their parents. These first promising results enrich the literature on front-of-packaging nutrition labels, providing insights on youth, for whom this topic has been little explored so far.

With almost all adolescents having already asked their parents to buy a particular food product and more than half buying at least occasionally food or beverages on their own, this study confirms that they act both as prescribers and buyers. They therefore constitute an interesting target for nutritional prevention actions and in particular the implementation of simplified labeling on the front of packaging.

With 97% of awareness in October 2021, almost all adolescents have already seen or heard of Nutri-Score. In addition, 9 out of 10 reported that the logo is easy to understand and even more easy to identify on food packages. These results are in line with studies conducted among adults in France, showing that the Nutri-Score logo, compared with other labeling systems, is considered the easiest to understand and to identify on packaging [28].

Moreover, these data are consistent with the results of a study conducted in Australia, which showed that the gradual logo health star rating (HSR) was preferred by young people (10–17 years) and adults, whereas the reference intakes were the least preferred. The main reasons given for this were that the HSR logo was easy to use, had interpretative content and was easy to spot [29]. In line with these data, another study found that the HSR was the most effective logo, compared with the multiple traffic lights and the reference intakes, to help young consumers identify the healthiest alternative among four products [30].

Overall, 70% of the French adolescents reported that they had already purchased a product with the Nutri-Score. For more than half who bought a product with the logo (54%), the Nutri-Score influenced the act of purchase. This can be explained by the fact that it is easy to identify and understand; it can therefore be used in a context of grocery shopping where choices are made relatively quickly [31]. More specifically, 47% have already opted for a product with a logo instead of a product without a logo and 47% have already changed a product or brand to buy a product with a better Nutri-Score. In a previous experimental study conducted among young people (7–11 years old) in France and relating to the Nutri-Score, the use of the logo was evaluated in a context of choice and showed effective results. The children (and their mothers) had to choose a snack among alternatives of different nutritional quality before and after the Nutri-Score was applied. The addition of the logo led to choices of better nutritional quality, confirming that, even at an age below our study population, the logo can be integrated among the many determinants of food choices [32].

In total, 7 out of 10 adolescents who know the Nutri-Score declared that their parents take the logo into account when making food purchases. In the previous study, the improvement in the nutritional quality of the snack chosen by the mothers also revealed that the latter had taken the logo into account when making their choices [32]. Overall, in our study, it appeared that parents were more likely to agree to buy a product that the adolescents requested because it had a more favorable Nutri-Score (A or B) (for 6 out of 10 young people knowing of the logo) rather than refusing a purchase because of the presence of a less favorable Nutri-Score (D or E) (3 out of 10). This is consistent with the results of the large scale randomized controlled trial conducted in 2016 in France. These have indeed shown that the improvement in the overall nutritional quality of the shopping basket linked to the Nutri-Score had mainly been induced by an increase in purchases of products of good nutritional quality rather than a decrease in purchases of products of poorer nutritional quality [8]. This is also in line with results reported in 2022 from shopping panels showing a higher progress on food sales for products with Nutri-Score A and B (+0.3 and 0.4, respectively), whereas the sales of products with Nutri-Score E decreased by 0.5 points [30]. This could be related to an evolution of the consumer choices but also of the food offer, with 3.5% less products displaying a Nutri-Score E in one year [33].

Results from the multivariate logistic model showed that the awareness of the Nutri-Score is significantly associated with gender and age; the girls and the 15–17-year-olds being more likely to be aware of the logo. However, interestingly, no significant difference was found for the occupational category of the reference person of the household, whereas a previous study performed in French adults reported that individuals from lower categories were less likely to be aware of the label [34]. One hypothesis to explain this discrepancy could be that these data were collected from 2018 to 2019, and since this period, the number of products displaying the logo significantly increased and different promotion campaigns of the Nutri-Score were broadcast. The gender-related differences can be explained by women’s greater interest in nutrition, often reported in the literature [31,35]. As regards age, even if the model was adjusted for buying products alone, the higher awareness of the 15–17-year-olds might be due to the fact that they are overall more involved in food purchase and preparation on their own. 

As regards behaviors, the most significant effects were observed among adolescents whose parents have taken the Nutri-Score into account during their grocery shopping. These results are consistent with data of the literature on the importance of parental practices on children’s eating behaviors [36]. Again, interestingly, no difference related to the occupation of the household reference person was observed. This is consistent with the results observed in adults and confirmed by a multivariate analysis showing that the logo use was independent of socio-professional category [34].

To our knowledge, this study is the first to report Nutri-Score results in adolescents. Nevertheless, certain limitations inherent to the survey methodology are worth recalling. First, the quota sampling method, which consists of interviewing the first people who respond to the survey solicitation, may tend to limit the response of the hardest-to-reach individuals, who are often the furthest from the recommendations. This method can thus lead to significant differences with samples selected by a random method [37], where respondents are randomly selected from a sampling frame and contacted individually, with a high insistence coefficient to increase the participation rate. Then, the questionnaire was administered online via an access panel. Although this method has advantages, such as cost or speed, it is likely to create a selection bias excluding people without internet access. In 2019, in France, 10% of households did not have access to the internet [38]; this situation was associated with older age, a lower level of education and a lower standard of living [39]. In addition, purchasing behaviors have been self-reported and have not been observed in real conditions. A social desirability bias may therefore have led to overestimating the impact on behavior. Finally, the study did not use validated measurement tools. Nonetheless, the questionnaire included some questions already used in previous studies [28,34] and was critically reviewed by the research team involved in this study. 

## 5. Conclusions

This first study on the awareness, perceptions and self-reported use of the Nutri-Score among French adolescents highlights a good appropriation of the logo by the 11–17-year-olds. Indeed, almost all reported to be aware of the label and consider it easy to understand and easy to identify on food packages. More importantly, 54% self-reported they have already been impacted by the Nutri-Score during their food purchase. This figure appears all the more important given that the logo is displayed on a voluntary basis and therefore in June 2021, about 57% of the sale volumes affixed the Nutri-Score on the French food market [40].

In addition, our data confirmed that adolescents acted both as buyers and prescribers; therefore, they could play a substantial role in food choices and, for this reason, they represent an interesting target for prevention campaigns on FOP nutrition labels. Nonetheless, our results also demonstrated the crucial role of parents as models for the transmission of food behavior, since the use of the logo by parents showed the higher odds of changing behaviors due to the Nutri-Score among adolescents. Finally, these data are also of importance given the extension of the Nutri-Score to other food environments such as food catering, where adolescents are also likely to be impacted by the logo. Further studies would be warranted to explore whether the Nutri-Score allows adolescents to improve their food choices in this specific context.

## Figures and Tables

**Table 1:** Individual characteristics of the participants (*n* = 1201-weighted data).

	Total		11–14 Years		15–17 Years		*p*-Value *
	*n* = 1201		*n* = 686		*n* = 515		
	*n*	%	*n*	%	*n*	%	
**Gender**							**0.0032**
Boys	614	51.1	376	54.8	238	46.2	
Girls	587	48.9	310	45.2	277	53.8	
**Occupational category of the reference person in the household**							0.50
Upper category	588	49.0	330	48.1	258	50.1	
Lower category/Inactive	613	51.0	356	51.9	257	49.9	
**Perception of the weight status**							0.68
Too skinny	95	7.9	58	8.5	37	7.2	
Normal weight	1001	83.4	561	81.8	440	85.4	
Overweight	105	8.7	66	9.7	38	7.4	

* *p*-value based on Chi-square test. Boldface indicates statistical significance (*p* < 0.05).

**Table 2:** Purchasing practices of adolescents on foods and beverages (*n* = 1201–weighted data).

	Total		11–14 Years		15–17 Years		*p*-Value *
	*n* = 1201		*n* = 686		*n* = 515		
	*n*	%	*n*	%	*n*	%	
**How often do you…**							
**…ask your parents to buy a specific food or beverage product?**							0.22
Often	456	38.0	248	36.1	209	40.5	
Sometimes	677	56.4	400	58.4	276	53.7	
Never	68	5.6	38	5.5	30	5.8	
**…go for food purchases with your parents or another person?**							0.83
Often	184	15.3	105	15.3	78	15.2	
Sometimes	771	64.2	442	64.4	329	63.9	
Never	247	20.5	139	20.3	108	20.9	
**…buy alone foods or beverages?**							**<0.0001**
Often	130	10.8	43	6.2	87	16.9	
Sometimes	538	44.8	250	36.4	288	55.9	
Never	533	44.4	393	57.3	140	27.2	

* *p*-value based on Chi-square test. Boldface indicates statistical significance (*p* < 0.05).

**Table 3:** Awareness, perception and purchasing practices related to the Nutri-Score label (weighted data).

	Total		11–14 Years		15–17 Years		*p*-Value *
	*n*	%	*n*	%	*n*	%	
	*n* = 1201		*n* = 686		*n* = 515		
**Awareness**							
Heard in the past	1031	85.9	566	82.5	466	90.4	**0.0001**
Visual recognition	1156	96.3	652	95.0	504	97.9	**0.0088**
Total awareness	1165	97.0	657	95.8	508	98.6	**0.004**
**Perception**							
Easy to understand	1093	91.0	618	90.1	475	92.2	0.22
Easy to identify	1114	95.7	624	94.9	491	96.6	0.16
	***N* = 1165**		***N* = 657**		***N* = 508**		
**Have ever bought a product with Nutri-Score**	836	71.8	440	67.0	396	77.9	**<0.0001**
**The Nutri-Score was an incentive to buy the product ^1^**	449	53.8	217	49.4	232	58.6	**0.008**
**Impact on behavior**							
Choosing a product with the Nutri-Score instead of a product without the label	549	47.1	280	42.6	269	52.9	**0.0005**
Choosing a product or a brand with a better Nutri-Score (e.g., D instead of E, or C instead of D)	543	46.6	280	42.7	263	51.7	**0.0022**
Asking your parents to buy a specific product because the Nutri-Score was affixed on it	173	14.9	89	13.6	84	16.6	0.15
At least one change in the purchasing behavior	630	54.1	331	50.4	299	58.9	**0.004**
**Parents’ behavior related to the Nutri-Score**							
Your parents take into account the Nutri-Score for food purchase	818	70.3	449	68.3	370	72.7	0.10
Your parents have ever accepted to buy you a product because its Nutri-Score was A or B	713	61.2	382	58.2	331	65.1	**0.016**
Your parents have ever refused to buy you a product because its Nutri-Score was D or E	345	29.6	197	30.1	148	29.0	0.71

* *p*-value based on Chi-square test. ^1^ Question asked only among adolescents who reported they have already bought a product with Nutri-Score (*N* = 836). Boldface indicates statistical significance (*p* < 0.05).

**Table 4:** Associations of Nutri-Score awareness (model 1) and change in purchasing behavior (model 2) with individual characteristics, based on logistic regression model (*n* = 1165).

	Awareness		Change in Purchasing Behavior	
	OR (95%CI)	*p*-Value *	OR (95%CI)	*p*-Value *
**Gender**		**0.028**		0.25
Boys	1		1	
Girls	2.28 (1.09–4.77)		1.16 (0.90–1.51)	
**Age**		**0.0094**		0.073
11–14	1		1	
15–17	3.12 (1.32–7.35)		1.28 (0.98–1.68)	
**Occupational category of the reference person in the household**		0.73		0.48
Upper category	1		1	
Lower category/Inactive	0.89 (0.45–1.74)		1.10 (0.85–1.42)	
**Perception of the weight status**		0.42		**0.030**
Normal weight	1		1	
Too skinny	3.63 (0.47–28.20)		0.56 (0.35–0.89)	
Overweight	0.81 (0.29–2.27)		1.20 (0.75–1.92)	
**Buy alone foods or beverages**		0.82		0.15
No	1		1	
Yes	1.09 (0.55–2.16)		1.22 (0.93–1.60)	
**Parents take into account the Nutri-Score for food purchase**				**<0.0001**
No	NA		1	
Yes	NA		7.74 (5.74–10.42)	

* *p*-value based on logistic regression models adjusted for gender, age, occupational category of the reference person in the household, perceived weight status and buying food alone or not. The model on behaviors was also adjusted on the use of the Nutri-Score by the parents. NA: the question of the use of the Nutri-Score by parents was only asked to adolescents who answered to be aware of the label. Boldface indicates statistical significance (*p* < 0.05).

## Data Availability

The data presented in this study are available on request from the corresponding author.

## Supplementary Materials

The following supporting information can be downloaded at: https://www.mdpi.com/article/10.3390/nu14153119/s1, Supplementary material 1: Questionnaire assessing Nutri-Score’s awareness, perceptions and self-reported use among French adolescents (11–17 years old) (November 2021).
