# Supplementary material for: Nutri-Score: Awareness, Perception and Self-Reported Impact on Food Choices among French Adolescents
Source: Nutrients. 2022 Jul 29;14(15):3119. doi: 10.3390/nu14153119 (PMC9370257; doi:10.3390/nu14153119)
Supplement: Supplementary file 1 [file nutrients-14-03119-s001.zip › nutrients-1822296-supplementary.pdf]

Supplementary material 1: Questionnaire assessing Nutri-Score's awareness, perceptions and self-reported use among French adolescents (11-17 years old) (November 2021)

The questionnaire was administered online to an access-panel implemented by a marketing survey firm specialized in opinion polls, BVA Group.
The original version was in French and translated in English for publication.

**Individual information and quotas**

Z2a. How old are you?

Z2b. Do you have a child between 11 and 17 years old?
1. Yes
2. No

If no: end of the questionnaire
If yes:
This questionnaire must be completed exclusively by an adolescent between 11 and 17 years old. If necessary, as a parent, you can help him complete the first questions that follow concerning his family situation and your profession.

Z1. You are…
1. A boy
2. A girl

Z2. How old are you?

Z0a. Please enter the zip code of the town where you live

Z0b. In which town do you live? *(automatic answer proposed according to the zip code)*

Z4. Most of the time you live...
1. with your father AND your mother
2. with your father OR your mother (or your legal guardian)
3. you do not live with your parents (or your legal guardian)

Z8. At the moment, what is the main activity of [Z4b]?
1. Employee
2. Self-employed
3. Looking for a first job
4. Looking for a job (has already worked)
5. Retired
6. At Home
7. Pupil or student
8. In another situation

if Z8=1 or 2
Z8b. What is the profession, the socio-professional category of [Z4b]?

If Z8=4

Z8b. Before being unemployed, what was the last occupation, the last socio-professional category of [Z4b]?

1. Farmer operator
2. Artisan small trader
3. Company manager with more than 10 employees
4. Liberal profession (EXCEPT paramedic)
5. Professor / scientific profession
6. Executive and other higher intellectual profession
7. Foreman, supervisor, paramedical profession, technician
8. Teacher
9. Employee
10. Service personnel
11. Laborer / Farm Laborer
12. Retired
13. Pupil/Student
14. Other Inactive

Z10. Currently you are...

1. In primary school
2. In middle school
3. In high school (excluding preparatory class)
4. In apprenticeship (CAP, BEP)
5. Student, in higher education (university, IUT, BTS, Prépa…)
6. You work
7. You are looking for a job
8. In another situation

From here, the following questions should be completed by the adolescent. In order not to influence his/her answers, we would be grateful if you would let him/her answer this questionnaire alone.

Food behaviour and Nutri-Score's awareness, perception and self-reported use

1. Do you ever…

a) cook or help to prepare meals at home
b) go for grocery shopping with your parents or another person
c) ask your parents or someone else to buy a particular food or drink
d) buy food or drinks alone

1. Yes, often
2. Yes, once in a while
3. No, never

2. Have you ever heard of the Nutri-Score logo, even if only in name?

1. Yes and I can see what it is
2. Yes, but I'm not sure what it is
3. No

3. Here is the Nutri-Score logo. Have you ever seen this logo?

Display of the logos (possibility to scroll through the 5 logos with different letters)

1. Yes
2. No

4. Indicate whether or not you agree with the following statements about this logo.
Random Item Rotation
If has already heard of or seen the logo (Q2 = 1 or 2 OR Q3 = 1), ask Q4b
a) This logo is easy to understand
b) This logo is easy to identify on food packages

1. Fully agree
2. Somewhat agree
3. Somewhat disagree
4. Fully disagree

If has heard of or seen the logo (Q2 = 1 or 2 OR Q3 = 1)
5. Have you personally ever bought food products or beverages with the Nutri-Score logo on it?
Display of the logos

1. Yes, often
2. Yes, sometimes
3. No never
4. I did not see the logo during my food purchase
5. I do not know anymore

If has bought a product with Nutri-Score logo (Q5 = 1 or 2)
6. You have already bought food products or beverages with the Nutri-Score logo on it. Did having this logo on the packaging influence your purchase?
If you have already purchased several products displaying this logo, please answer in relation to your last purchase.
Display of the logos

1. Yes
2. No

If has heard of or seen the logo (Q2 = 1 or 2 OR Q3 = 1)
7. Did the logo ever…
Random Item Rotation
a) make you choose a food/beverage on which there was this logo, rather than another product without a logo
b) make you change of product or brand to buy a food/beverage with a better Nutri-Score (for example D rather than E or C rather than D)

1. Yes, often
2. Yes, sometimes
3. No, never

If has heard of or seen the logo (Q2 = 1 or 2 OR Q3 = 1)
8. Have you ever asked your parents to buy you food products or beverages precisely because it had this logo on the packaging?

1. Yes
2. No

If has heard of or seen the logo (Q2 = 1 or 2 OR Q3 = 1)
9. Do your parents take into account the presence of this logo on the packaging when making their food purchase?

1. Yes, often
2. Yes, sometimes
3. No, never
4. I don't know

If has heard of or seen the logo (Q2 = 1 or 2 OR Q3 = 1)

10. Have your parents ever ACCEPTED to buy you food products or beverages that you asked for because it had a Nutri-Score A or B?

1. Yes
2. No

If has heard of or seen the logo (Q2 = 1 or 2 OR Q3 = 1)

11. Have your parents ever REFUSED to buy you food products or beverages you asked for because it had a Nutri-Score D or E?

1. Yes
2. No

Other information

12. Currently, you consider yourself to be…

1. Too skinny
2. Normal weight
3. Too big

13. Currently, do you have a special diet?

Several possible answers except "no"

1. Yes, to lose weight or not to gain weight
2. Yes, to gain weight
3. Yes, because of a food allergy or intolerance
4. Yes, for another medical reason
5. Yes, vegetarian, vegan or vegan
6. Yes, by religious conviction
7. No
